# Healing with Herbs: A Systematic Review of Natural Treatments for Polycystic Ovary Syndrome

**DOI:** 10.5935/1518-0557.20240110

**Published:** 2025

**Authors:** Rashmi Wani, Mushtaque Shaikh

**Affiliations:** 1 Department of Pharmaceutical Chemistry, Vivekanand Education Society’s College of Pharmacy Affiliated to University of Mumbai, Chembur, Mumbai-400074, India

**Keywords:** PCOS, hyperandrogenemia, insulin, herbal, testosterone

## Abstract

**Objective:**

With one in ten women globally suffering from it, polycystic ovarian syndrome (PCOS) has recently emerged as one of the most common endocrine multifactorial illnesses. Each patient may not experience all the potential symptoms of PCOS, which include insulin resistance, hirsutism, acanthosis nigricans, hyperandrogenism, weight gain, etc.

**Methods:**

Although symptomatic treatments like ovarian drilling procedures or cosmetic lotions to alleviate hirsutism do not address the underlying cause, there is still no comprehensive treatment for PCOS. It is important to take into account the psychological and occupational factors influencing the unfavourable rise in infertility.

**Results:**

Changes in lifestyle, nutrition, prolonged diabetes medication, oral contraceptive pills, and ultimately laparoscopic surgery are obstacles to treating the syndrome. To lower the cost, time, and side effects of current treatments, polyhedral formulations must be developed in light of the aforementioned aspects. Since natural resources are being utilized widely, classes of compounds like coumarins, flavones, lignans and terpins have recently drawn the attention of researchers as a potential source of medication for therapy.

**Conclusions:**

This article suggests a list of herbal medications that, when combined with other PCOS treatments, can be successful. These herbs may also be sufficient on their own to treat ovarian cysts naturally.

## INTRODUCTION

Polycystic ovary syndrome (PCOS) is a complex condition involving multiple symptoms simultaneously in patients and primarily affects women aged 18 to 44. An entity otherwise known as Stein-Leventhal Syndrome PCOS is sparked by the evident anomalies that have served as metabolic syndromes-obesity, dyslipidemia, hypertension, atherosclerosis gestational diabetes, risk of type II diabetes, and chronicity as well as incidence of infections of the respiratory tract with cough and cardiovascular disease (Allahbadia & Merchant, 2008). Hyperandrogenism, a biochemical hallmark of PCOS, is an elevated level of androgens marked in females primarily responsible for PCOS, which includes cutaneous indicators such as male pattern baldness, alopecia, and hirsutism, which results in disturbances in GnRH pulses, giving rise to an increased level of LH/FSH ratio (Goswami *et al.*, 2012).

The increased testosterone levels and their effect are a consequence of the concurrent overproduction of testosterone and decreased levels of sexual hormone-binding globulin (SHBG) seen in PCOS. Both of these effects have been speculated as consequence of excessive insulin. During menstruation, intake of high-calorie foods such as milk, fruits, and starchy foods leads to disrupting the organ’s blood flow, resulting in obesity, abdominal pain, and eventually conception failure (Franks *et al.*, 2006; Rooney & Pendry, 2014). One possible cause of the disease is reduction in levels of aromatase enzyme that converts testosterone to estrogen. The true prevalence of PCOS is unknown, while it ranges widely from 2.2 to 26% over the world. According to Rotterdam’s criterion, in Indian scenario, South India and Maharashtra had prevalence rates of 9.13 and 22.5%, respectively. India being rich in knowledge of herbal medicine and resources, leads us to think about this factor behind the wide difference in occurrences of PCOS in these two regions. To this purpose, the current study aims to determine how well natural chemicals and medicinal plants function to treat polycystic ovaries.

## EPIDEMIOLOGY OF PCOS

**Figure 1:**
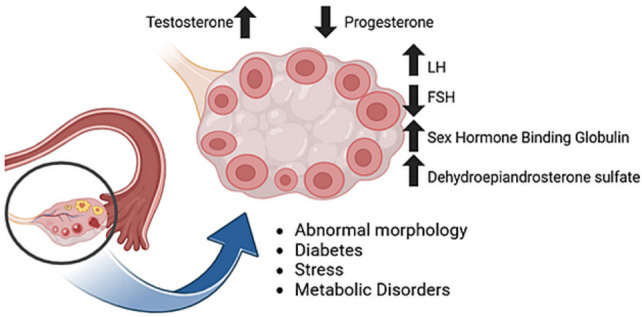
Schematic explanation of polycystic ovarian syndrome (Image generated using BioRender: https://app.biorender.com/).

One of the elements that contribute to PCOS is ethnic precedence and inherited. The pituitary gland secretes an irregularly high amount of luteinizing hormone (LH) into the bloodstream, disrupting a female’s normal menstrual cycle and eventually creating an imbalance between progesterone, estrogen, Follicle Stimulating Hormone (FSH), and (LH), which leads anovulation (Khanage *et al.*, 2019). The abnormal amount of testosterone hormone in the ovaries prevents ovulation, resulting in infertility. As a result, cysts or fluid-filled sacs are formed, which are little more than an underdeveloped follicle that remains in an undissolved state, as seen in Figure 1.

**Figure 2:**
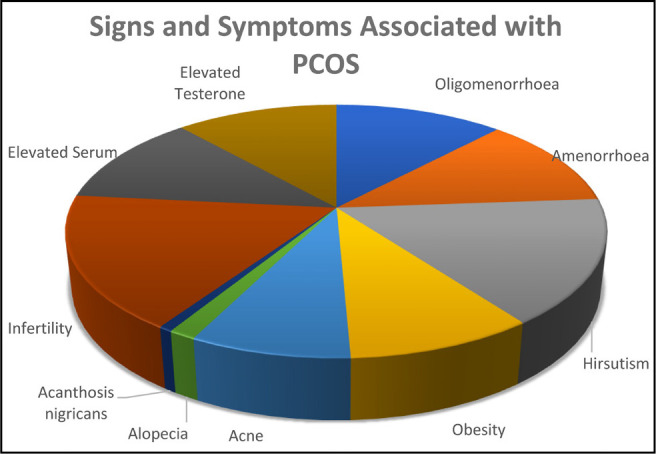
Signs and Symptoms associated with PCOS.

**Figure 3:**
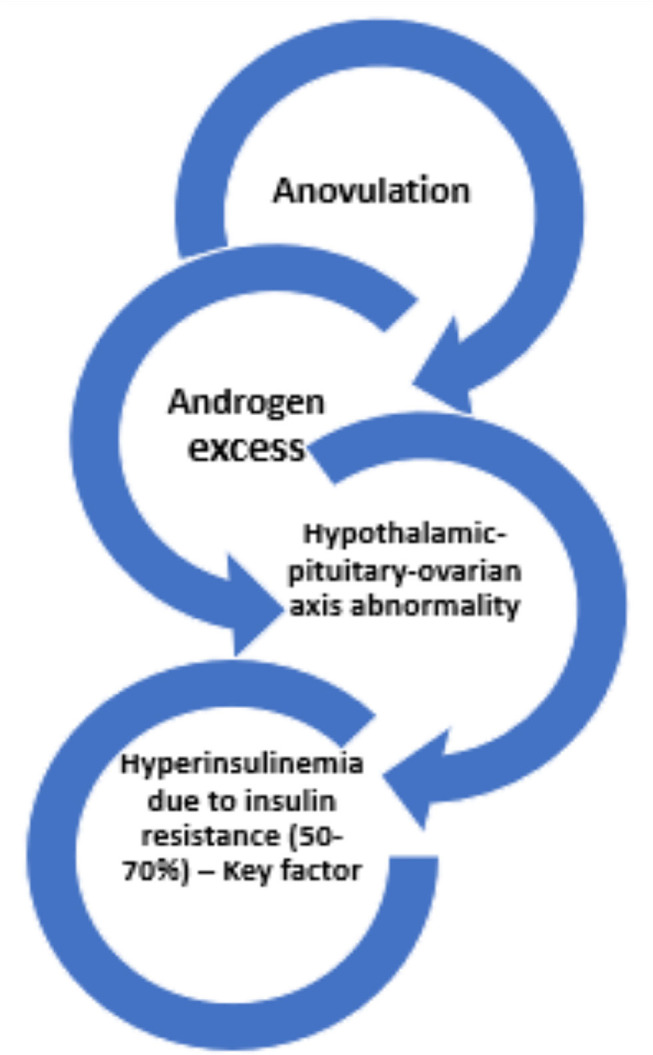
Factors responsible for PCOS.

Excess androgen causes acne, which is difficult to treat, hirsutism, androgenic alopecia/baldness, resulting in a cascade of events. Figure 2 displays PCOS-related signs and symptoms (Dunne & Slate, 2006). Diabetes, hypertension, heart disease, dyslipidemia, endometrial cancer, breast cancer, and recurrent loss of pregnancy are among clinical manifestations of this condition. PCOS patients have lower Melatonin concentrations in their follicles and experience moderate sleep problems. The causes and symptoms are delicately interlinked as depicted in the Figure 3.

## EFFECTIVE MEDICINAL PLANTS FOR THE MANAGEMENT OF PCOS

### Ashwagandha

*Withania somnifera* (WS), or Indian ginseng, commonly known as ashwagandha, belongs to herbs that have been used in Ayurvedic medicine for over 2500 years. Many investigations have confirmed its use in health benefits as antistress, antibacterial, anti-inflammatory, antioxidant, and immunomodulatory agents. In addition, it has been shown to be clinically effective in Parkinsonism, anxiety, and insomnia conditions and also showed supplementary therapeutic effects in patients undergoing radiation and chemotherapy treatments. In their experiments, WS raised cortisol in blood, increased the ability of mice to tolerate and resist stress, and reduced fatigue. Anwer and co-workers found that WS significantly improved the glycemic control and insulin sensitivity of non-insulin-dependent streptozotocin diabetics. Ashwagandha has been used worldwide as a nutritional supplement (Budhiraja & Sudhir, 1987). The key biological agent responsible for its therapeutic activities has been identified as steroidal lactones, sometimes referred to as withanolides, due to their diverse biological properties (Santhi & Aishwarya, 2011). One study has been recorded that use of ashwagandha in a patient suffering from PCOS. A 57-year-old woman was reported been experiencing significant scalp hair loss, dryness, and a burning feeling in her scalp. She did not have any problems about her reproductive system, hirsutism, menstruation, obesity, or acne. Her BMI was 21.5 kg/m^2^. Her daughter was diagnosed with PCOS. The patient was advised to take 400 mg ashwagandha twice daily since it has anti-stress and antioxidant effects. At this dose, she initiated standardized root of ashwagandha. Hair fall reduced to almost normal level within a span of one month. Equally tested were levels of corticosterone, cortisol, and 11-deoxycortisol in blood. Serum levels of 18-hydroxycorticosterone, 17-OH-pregnenolone, corticosterone, and 11-deoxycortisol have fallen (Kalani *et al.*, 2012).

### Fenugreek

*Trigonella foenum-greacum L,* also known as fenugreek, is a natural medicinal plant. Fenugreek seeds have been demonstrated to be hypocholesterolemic and anti-diabetic in both human and animal studies. There are no toxicological effects associated with the administration of fenugreek. Furthermore, Shamas et al. concluded that fenugreek is safe to use. The goal of this study was to determine the effect of fenugreek seed extract (*Trigonella foenum-greacum L*.) on insulin resistance in women with PCOS. The following investigations were carried out to investigate the effect of Fenugreek on PCOS.

1. The Montaserieh Hospital of Mashhad University of Medical Sciences in Iran undertook a double-blind, prospective, randomized, placebo-controlled trial. The study included 58 women with normal ovaries who had oligo-anovulatory PCOS. Over the course of an 8-week treatment, women were randomly assigned to receive either hydroalcoholic extract of fenugreek seeds with metformin capsules (n=30) or placebo capsules with metformin (n=28). The participants underwent assessments before and during each four-week period. Furocyst, a new fenugreek seed extract, has been shown to reduce ovarian volume and the number of ovarian cysts in women with PCOS. It also resulted in the regularization of menstruation cycles, and some participants became pregnant. Overall due to this administration of Fenugreek as a medicine daily, majority of the women were able to restore their menstruations and some their fertility (Hassanzadeh Bashtian *et al.*, 2013).

2. In a study of 50 premenopausal women (18-45 years old, BMI<42) with PCOS, a novel *Trigonella foenum-graecum* seed extract (fenugreek seed extract, Furocyst, 2 capsules of 500 mg each/day) enriched in about 40% furostanolic saponins was used in an open-label, one-arm, non-randomized post-marketing surveillance study over 90 consecutive days. The study sought to determine its efficacy in reducing the number of ovarian cysts and ovarian volume. Treatment with furocyst led in a significant decrease in ovarian volume. Approximately 46% of the study participants had a reduced cyst, whereas 36% had a fully dissolving cyst. It is noteworthy to note that due to the following therapy, 71% of participants reported to have a regular menstrual cycle again, and 12% of participants were able to conceive. In total, the regimen was beneficial to 94% of the patients. Comparing the results to the baseline, luteinizing hormone (LH) and follicular stimulating hormone (FSH) levels showed significant increases. Broad-spectrum safety was proven by numerous haematological, biochemical, and blood chemistry tests. Both the volume of the ovaries and the number of ovarian cysts were significantly reduced with furocyst. Fenugreek has had a great impact on the patients of PCOS. Due to its medicinal advantage, it has reduced the ovarian volume and has restored quite a few things, such as menstruation which are essential in the life of women (Swaroop *et al.*, 2015).

### Shatapushpa-Shatavari

Known by the common name Shatavari, *Asparagus racemosus* is widely distributed in Sri Lanka, India, and the Himalayas. The most prevalent application for Aracemosus is in traditional medicines. It includes essential fatty acids, polycyclic alkaloids, isoflavones, flavanoids, and steroidal saponins, also referred to as shatavarin. In clinical Ayurvedic practices, Satapushpa Shatavari powder (SSP) and/or Satapushpa-Shatavari Grita (SSG) Matra Vasti (therapeutic enema) are frequently used as a treatment for polycystic ovarian syndrome (PCOS)-related menstruation symptoms.

However, no conventional clinical research study has examined its efficacy clinically. In Ayurveda and traditional medicine in Sri Lanka, Satapushpa (*Anethum graveolens*) and Shatavari (*Asparagus racemosus*) are used for conditions of oligomenorrhea, hypomenorrhoea, and an ovulation; furthermore, it has been noted that this drug can be used in oral and/or enema and/or nasal instillation. Attempt to assess the clinical efficacy of oral root administration and per rectal administration of Satapushpa - Shatavari formula on PCOS patients had been reported in the literature.

For the study, the PCOS premenopausal female patients were selected which were diagnosed with PCOS as per Rotterdam criteria and aged between 18-42-year. The Ultrasound scan’s criteria for evaluating patients both before and after therapy included the development of PCOS in the ovaries, their volume, endometrial thickness, body weight, quantity of menstrual blood per cycle, duration of menstruation and hirsutism rating score. Precedence of following two studies are found.

1) The 60 participants in the study were randomly assigned to test groups A (SSP oral), B (SSP oral plus SSG enema), and C (SSG enema). The researchers combined all other patients into one group. Every patient spent one month in the test group and three months in the follow-up group. Three patients from group C, two from test group A, and one patient from test group C all voluntarily stopped participating. Thus, 54 subjects were included in the analysis. Following a one-month study period, the groups’ endometrial thickness, ovarian volume, and symptooms of PCOS were measured using a US scan. Every patient was instructed to return for follow-up appointments every 14 days for a 2-month period.

Results were found on reducing the volume of the ovary measured by ultrasound scan (US) in all three groups. The study found that all three groups’ endometrial thickness had improved, with group C demonstrating a substantial (*p*<0.05) increase in endometrial thickness when compared to the other two groups. The drug-treated group C’s hirsutisum scores showed a considerably lower result in the trial. Quantity of the menstruation of drug treated groups A (*p*<0.05), B and C (*p*<0.01) were significantly improved. The length of each group’s menstrual cycle was longer when they received medication (Kumarapeli *et al.*, 2018).

2) In another study, females of reduced fertility level of 25-40 years age group were selected. Patients suffering from diabetes mellitus, hypertension, thyroid disorders, hyperprolactinemia and congenital adrenal hyperplasia, other gynaecological disorders, heart diseases and renal failure were excluded from the study. Over the course of six months, the therapy was administered in three parts. Patients in stage 1 (Days 1 to 14) received 30ml of Triphala Kwatha, 2 Chandraprabha pills, and 5g of Manibhadra powder twice a day at 6 AM in the morning and evening. These medications were given to the patients to perform body cleansing, and they are all beneficial to the function of the female genital organs.

Patients were treated with 5g of powdered Shatavari (*Asparagus racemosus*), Shatapushpa (*Peucedanum graveloens*), and Guduchi (*Tinospora cordifolia*) administered twice a day in the morning and evening during stage 2 (Days 15 to 4^th^ month). Additionally, they received treatment twice a day in the morning and evening with 20ml of Krishna Jeeraka. Stage 3 (4th to 6th month) patients were given 4 pills (each 125mg) of Rasayana Kalpa (*A. racemosus, Terminalia chebula, T.belarica, Embelica officinalae, T. cordifolia, Naredostachys jatamansi, Herpestis monnieria*) twice a day at 6 PM along with 5g of each powder of Atibala (*Abutilon indicum*) and Shatapushpa. They were also given 20 millilitres of Sahachara oil twice a day, at 8 am in the morning and evening. These patients received local treatment with Uttara Vasti, which involves a combination of 5ml of Shatapushpa oil with water, for two consecutive days each month starting on the day when the menstrual flow entirely stops, usually between the fourth and tenth day of the cycle.

Only 25% of the patients were found to have any irregularities in their menstrual cycle at the completion of the treatment. By the time the treatment was over, 75% of patients were free of dysmenorrhea, 57.5% of patients had normal monthly bleeding length, and 70% of the patients had average menstrual blood volume. When it comes to infertility caused by polycystic ovarian syndrome, 75% of patients were conceived while 85% of patients were effectively treated for the condition (Dayani Siriwardene *et al.*, 2010).

### Maitake Mushroom

Strong immune-stimulating properties of maitake mushrooms (*Grifola frondosa*) have been reported, and an active extract known as “D-fraction” has been found. Additionally, studies have shown that maitake mushrooms can lower blood pressure and blood glucose, and modify serum lipid levels (Fukushima *et al.*, 2001; Horio & Ohtsuru, 2001; Kabir *et al.*, 1987; Konno *et al.*, 2001; Kubo *et al.*, 1994; Manohar *et al.*, 2002; Talpur *et al.*, 2002). Recently, a novel bioactive extract known as “SX-fraction” (MSX), which is a water-soluble glycoprotein with an average molecular weight of 20,000 Da, was discovered to be an extract that can improve insulin resistance.

An open trial was conducted at three clinics in Japan involving eighty PCOS patients by Chen *et al.* (2010). For a maximum of 12 weeks, seventy-two (72) newly admitted patients were randomised to receive either MSX or Clomiphene Citrate (CC) monotherapy. For up to 16 weeks, 18 patients who did not respond to either MSX or CC received combination therapy consisting of both drugs. From the start, 8 patients who had a history of proven failure to respond to CC were treated with combination therapy. Ultrasonography was used to measure ovulation. Ovulation was assessed in 26 participants in the MSX group and 31 patients in the CC group. According to the patients (NS), the ovulation rates for MSX and CC were 76.9% (20/26) and 93.5% (29/31), respectively, while the cycles showed 69.9% (58/83) and 41.7% (30/72), respectively (*p*=0.0006). Seven out of the seven patients who failed MSX monotherapy and six out of the eight patients who failed CC alone had ovulation in the combined medication. Of the three subjects in this study, some became pregnant after receiving MSX medication. Overall, Maitake Mushroom was found very productive in reducing blood pressure, blood glucose and modifying serum lipid levels (Chen *et al.*, 2010).

### Sesame Oil

The oil seed sesame (*Sesamum indicum L.*) is a significant crop in Indian subcontinent and numerous health benefits of the seeds have been reported. Sesamin, a bioactive substance obtained from sesame, shields the liver from oxidative damage. Additionally, it has been discovered that sesame seeds contain inherent antibacterial properties against common skin pathogens like Streptococcus and Staphylococcus species, in addition to anti-viral, anti-fungal, and anti-inflammatory properties (Anilakumar *et al.*, 2010). Sesame seeds have been shown in earlier research to possess flavonoids and other phenolic compounds with potential antioxidant properties (Zhou *et al.*, 2016). There were no reports on human trials but in case of animal study in vivo, twenty-eight nonpregnant female Wister albino rats were used in the study, and they were split into four equal groups. Sesame oil was found to be having a mitigative effect on PCOS. It showed reduction in the ovarian cysts and reduced the male hormone secretion in the female patients and eventually increased progesterone level (Elshamy *et al.*, 2023).

### Chamomile

Research on chamomile extract has revealed that the plant’s medicinal properties are mediated by both hydrophilic and lipophilic components. This plant species is mostly known for its volatile oil, sesquiterpene lactones, and phenols, which include flavonoids. Numerous phenolic chemicals, mainly flavonoids, apigenin, quercetin, patuletin, luteolin, and their glucosides, are the major components of chamomile flowers. All vascular plants contain flavonoids, which are chemical phenyl benzopyrones that are typically conjugated with sugars. Flavonoid natural products have a molecular scaffold known as the benzopyranone ring system, which exhibits modest aromatase inhibitory action (Brueggemeier *et al.*, 2001). The subgroup of flavone, which includes luteolin, apigenin, and flavone all three of which are found in chamomile is one of the six major subgroups of flavonoids. Roman chamomile, also known as Chamaemelum nobile, is a perennial herb that is grown in Western Europe and North Africa. It is a synonym for *Anthemis nobilis L* of the *Asteraceae* family. Chamomile flowers are used in traditional medicine to make an anti-inflammatory and anti-spasmolytic tea for stomach problems. The antispasmodic properties of chamomile help women experience less painful menstrual cramps and lowers the risk of encountering a premature labour. Additionally, it has been discovered to induce menstruation (Maschi *et al.*, 2008). Because chamomile extract stimulates leukocytes (B lymphocytes and macrophages), it is used to treat eczema and skin irritations (Ziyan *et al.*, 2007).

Mature virgin cycling at thirty 200-220 g Wistar rats were split into two groups and placed in cages every six mice. Vaginal smears taken between 0800 and 1200 hours were used to track estrous cyclicity. Light microscopy was used to evaluate the relative amounts of leukocytes, epithelial cells, and cornified cells found in daily vaginal lavages, which varied depending on the stage of the estrous cycle. In both control and PCO rats, the rat estrous cycle (proestrus, estrus, metestrus, and diestrus) typically lasts four days. Flowers of chamomile were harvested from Ahvaz, Iran’s natural resources. The dried flowers were ground, and then 70% ethanol was used to repeatedly extract the plant components. To obtain a powdered extract, the fluid was filtered and vacuum-expelled. Human trial was conducted as follows. Thirty-Eight-week-old rats were split into two groups of control and PCO rats after a week of acclimatisation. To induce PCO, as per Brawer 1996, all rates assigned to the PCO group got an intramuscular injection of 0.2 mg of estradiol valerate (EV) (Aburaihan Co., Iran) in 0.2 ml of maize oil. The control group received 0.2 ml of maize oil. When follicular cysts were initially discovered, 60 days following injection, all the rats receiving EV treatment were assessed. The PCO rats were then split into four groups: three groups got varying amounts (25, 50, and 75 mg/kg) of alcoholic chamomile extract intraperitoneally for ten days, whereas the third group did not receive any extract. ELISA method was used to measure serum LH, FSH and estradiol levels. In addition to increasing dominant follicles, chamomile extract of dried *matricaria chamomill*a L. flowers can help rats recover from an induced PCO condition. This is likely because the GABA system interacts with chamomile’s effects on regulating LH surge secretion. It results in improved endometrial tissue configurations in the uterus (Farideh *et al.*, 2010).

### Black Kohosh

In the regions of Europe, Asia, Australia, and America, black cohosh (*Actaea racemosa* (AR), formerly known as *Cimicifuga racemosa*), is a popular herbal remedy for a range of conditions affecting women’s health (Bai *et al.*, 2007; Borrelli & Ernst, 2008; Burdette *et al.*, 2003; Mohammad-Alizadeh-Charandabi *et al.*, 2013). The hydroxycinnamic acids, caffeic acid, ferulic acid, phenolic chemicals, chromones, triterpenoids, and isoferulic acid, along with their condensation products with glycoloyl phenylpropanoids, also referred to as cimicifugic acids, are the main phenolic components of black cohosh (Nikolić *et al.*, 2015; Salari *et al.*, 2021). The study by Azouz *et al.* (2021) showed that, a combination of Actaea racemosa and vitamin C reduces PCOS’s metabolic and reproductive problems. With improved hormonal profile, lipid profile, glucose level, and liver functions, the AR arsenal of secondary metabolites countered ovarian oxidative stress, which may be involved in the development of PCOS, by inhibiting the androgen aromatization in the letrozole-induced PCOS rats. Moreover, AR or its combination with vitamin C led to a considerable downregulation of Cyp19-α1 mRNA expression level and an increase in Ki-67 expression in the granulosa cell layer. Further research on the combination of AR and vitamin C in polycystic ovarian syndrome is warranted, given the results of steroidogenesis regulation with a decreased risk of hepatic side effects (Azouz *et al.*, 2021).

### Spearmint

The chemical constituents naming around 57.02% carvone, 24.63% limonene, 2.7% pulegone, 1.8% menthol, and 0.34% cineole are found in the pure Mentha spicata essential oil.

The in vivo study conducted on rats that had two regular estrous cycles were weighed and labelled as Group I (control) to Group V wherein the group was given 1 millilitre of distilled water orally for 20 days; subsequently letrozole; letrozole and 150 mg/kg of spearmint oil; letrozole and 300 mg/kg of spearmint oil; letrozole and sesame oil; 150 mg/kg of spearmint oil; 300 mg/kg of spearmint oil; and the last group with sesame oil. Rats in groups II, III, IV, and V were given letrozole orally (1 mg/kg) thrice a day for 28 days to induce PCOS. This was verified by looking for signs of protracted estrus phase and ovarian cysts in ovarian histological sections. Rats were given oral doses of sesame and spearmint oils for a duration of 20 days. The PCOS-induced groups that were given spearmint oil had testosterone levels that were significantly lower (*p*<0.001) than the PCOS-induced group. Research has demonstrated that when PCOS women drink spearmint teas, their testosterone levels decrease. According to other research utilising spearmint extract, spearmint has no effect on body weight in normal conditions (Nozhat *et al.*, 2014). However, spearmint helps control body weight, reduces the number of Graafian follicles, primordial follicles, and corpus lutea in PCOS conditions.

### Flaxseed

Flaxseed (*Linum usitatissimum L.*) has been shown to alleviate PCOS symptoms due to its phytoestrogen content so it is used in the management of endocrine disorders and to control the female sex hormones (Nowak *et al.*, 2007). In a study, hydroalcoholic extract of spearmint and flaxseed were used, in contrast to the control group, the PCOS group had significantly higher levels of testosterone and estradiol, and much lower levels of progesterone. When compared to the PCOS group, the treatment group’s progesterone levels were found to be increased considerably, and this rise was marginally more than when utilising flaxseed or spearmint alone as a treatment (Jelodar *et al.*, 2018). It has been observed that giving hydroalcoholic flaxseed extract to young rats considerably raises progesterone levels. In comparison to the PCOS group, the T group had less cystic follicles, with an increase in the thickness of the granulosa layer and substantially decreased theca layer. Flaxseed is very efficient in alleviating PCOS symptoms (Mehraban *et al.*, 2020). Emam et al took the research on the work to next level by exploring action of oil from these seeds on molecular targets, and the oxidative response in hyperandrogenism-induced polycystic ovary with a 21 days long study on PCOS model rats. In this state of the art research expression ratio of the steroidogenic acute regulatory protein (StAR) and Cyp11A1 gene were evaluated. The study revealed encouraging effects like restoration of glutathione (GSH), malondialdehyde (MDA), beta subunit luteinizing hormone (LH), testosterone levels etc justifying its usefulness.

### Fagonia indica

Quercetin and Myricetin were discovered to be the main flavonoid phytoconstituents in *fagonia indica* which belongs to the family *Zygophyllaceae*. This family comprises of all species that are shrubs or shrublets and herbs (Beier, 2005). The common name for fagonia is dhamasa booti. It possesses properties such as laxative, antiulcerogenic, thrombolytic, antimicrobial, antidiabetic, antitumor, etc. Fagonia has been traditionally used for thousands of years to alleviate menstruation issues. For experimental purposes, rats with induced PCOS were given a *F. indica* semi solid extract. It was found that PCOS rats gained more weight than normal control rats. *F. indica* treatment increased FSH levels while lowered testosterone levels. According to the current study, taking F. indica for treatment almost brought LH levels back to normal. Overall fagonia indica is found very useful in alleviating PCOS symptoms (Younas *et al.*, 2022).

### Chickpea

Chickpea (*Cicer arietinum*) has been traditionally used in Greek and Indian medicine for hormonal issues like menstrual induction, labor acceleration, placenta treatment, and lactation stimulation. Isoflavones in chickpea sprouts show estrogenic activity and are thought to protect against hormonal and metabolic imbalances in women with polycystic ovary syndrome (PCOS).

In a study, by Ali and coworkers from Cairo University, 35 rats were divided into three groups as control, letrozole-induced PCOS models, treatment groups. The third group received either clomiphene citrate or chickpea sprout extract (CSE). After 28 days, ovarian and uterine weights, histopathological changes, antioxidant levels, and hormonal/metabolic profiles were assessed.

Analysis showed significant reductions in ovarian weight in the treatment groups. Cystic follicles decreased in number and size, granulosa cell thickness increased, and theca cell thickness decreased. Hormonal and metabolic profiles improved, along with antioxidant status, while Cyp11a1 mRNA expression was downregulated. This study suggests that chickpea sprout extract may improve reproductive and metabolic symptoms in PCOS, highlighting its potential therapeutic effects.

Table 1 summarizes herbs reported is of fruitful in the treatment of PCOS with permissible active constituents.

**Table 1:** List of herbs used in PCOS treatment with their role.

Name of herb	Botanical name	Family	Effective Constituent	Pharmacological activities
Ashwagandha	*Withania somnifera*	Solanaceae	withanolide	Stress levels are minimised by balancing Cortisol levels
Fenugreek	*Trigonella foenum-greacum L*	*Fabaceae*	furostanolic saponins (Furocyst)	Reduce Insulin resistance, Lowers the Cholesterol, reduce the size of ovarian cysts, Regularize the menstrual periods
Shatavari	*Asparagus racemosus*	*Asparagaceae*	shatavarin	Improves follicular maturity, Regulates Menstrual cycle, counteracts the influence of hormones
Shatapushpa	*Peucedanum graveloens*	*Apiaceae*	carvone, flavonoids	Follicular aturation promoter, menstrual cycle regulator, reduces insulin resistance
Maitake Mushroom	*Grifola frondose*	*Meripilaceae*	phenol, flavonoids, unsaturated fatty acids	Induce ovulation and in management of Diabetes
Sesame Oil	*Sesamum indicum*	*Pedaliaceae*	lignans, linoleic acid	Reduces ovarian cyst and balances mono and polyunsaturated fats
Chamomile	*Chamomilla recutita*	*Asteraceae*	flavonoids, apigenin, quercetin, patuletin, luteolin	Reduces Luteinizing hormone and Improves Ovarian Morphology
Black Cohosh	*Actaea racemose*	*Buttercup*	caffeic acid, ferulic acid, phenolics, chromones	Induce ovulation
Spearmint	*Mentha piperita*	*Lamiaceae*	carvone, limonene	Helps in follicular development, reducing level of Testesterone
Flax seeds	*Linum usitatissium*	*Linaceae*	linolenic acid, linoleic acid, lignans, cyclic peptides, polysaccharides, alkaloids,	Suppression of Testosterone levels and Hirsutism, rise in progesterone level
Fagonia indica	*Fagonia indica*	*Zygophyllaceae*	quercetin, myricetin	Alleviate menstrual problems
Chickpea	*Cicer arietinum*	*Fabaceae*	Isoflavones	menstrual induction, acceleration of parturation, treatment of retained placenta and stimulation of lactation

## CONCLUSION

Female infertility is caused by an endocrine illness called polycystic ovary syndrome (PCOS). Numerous studies have demonstrated the significant role that herbal medications with low side effects play in the management of PCOS, even though using herbal medications to treat PCOS takes longer compared to the chemical ones. What can be done with this pool of identifying actives is identifying the targets, planning new formulation and subsequently combination of semisynthetic drugs and lead optimization for the root cause cure. Herbs have been shown to improve the body’s immune system and regulate the period of menstruation without altering hormone levels, which explains their effectiveness in treating PCOS. Though it has been proved that some of the herbal remedies have been established as possible cure for PCOS, further studies at biochemical and molecular biology level is yet to establish the targets and mechanisms of these remedies. At time they need to used in combination and hence roots behind interdependence and synergism must be understood. Similarly detailed studies on safety and quality issues need to be understood in future. Similarly identifying the active constituents in solitude or combinations if identified it will be possible to make synthetic analogues with better pharmacokinetics and dynamics. Through, the formulation of these herbs and quantification of their standard, release, and bioavailability is another possible hurdle, considering these obstacles as opportunities, it is expected to be a significant scope for pharmaceutical discovery and development.
